# Danon disease: a case report and literature review

**DOI:** 10.1186/s13000-021-01100-8

**Published:** 2021-05-01

**Authors:** Jiamin Xu, Zhu Li, Yihai Liu, Xinlin Zhang, Fengnan Niu, Hongyan Zheng, Lian Wang, Lina Kang, Kun Wang, Biao Xu

**Affiliations:** 1 Department of Cardiology, Affiliated Drum Tower Hospital, Medical School of Nanjing University, Nanjing, 210008 P.R. China; 2 Department of Pathology, Affiliated Drum Tower Hospital, Medical School of Nanjing University, Nanjing, 210008 P.R. China

**Keywords:** Danon disease, Cardiomyopathy, *LAMP*2, Mutation, NGS

## Abstract

**Background:**

Danon disease (DD) is a rare x-linked dominant multisystemic disorder with a clinical triad of severe cardiomyopathy, skeletal myopathy, and mental retardation. It is caused by a defect in the lysosomal-associated membrane protein-2 (*LAMP*2) gene, which leads to the formation of autophagic vacuoles containing glycogen granule deposits in skeletal and cardiac muscle fibers. So far, more than 50 different mutations in *LAMP*2 have been identified.

**Case presentation:**

Here, we report an 18-year-old male patient who was hospitalized for heart failure. Biopsy of the left lateral femoral muscle revealed scattered autophagic vacuoles in the muscle fibers with increased glycogen. Next generation sequencing (NGS) was used to detect gene mutations of the proband sample and a novel frameshift mutation (c.1052delG) has been identified in exon 8 of *LAMP*2, which leads to truncation of the protein.

**Conclusion:**

We found a novel frameshift mutation, a hemizygous mutation (c.1052delG) in exon 8 of *LAMP*2, identified as presenting the hypertrophic cardiomyopathy (HCM) phenotype. Genetic analysis is the gold standard for the diagnosis of DD and is essential to determine appropriate treatment strategies and to confirm the genetic risk of family members.

## Background

DD is a rare x-linked dominant multisystemic disorder with clinical manifestations of severe cardiomyopathy, skeletal myopathy, and mental retardation. It was first described in 1981 [1], and the pathogenic defect of *LAMP-2* was identified 20 years later [2]. DD was initially thought to be caused by a glycogen storage defect of glycogen accumulation in lysosomes. However, recent studies have suggested that the underlying mechanism is blockage of autophagy [3], leading to impaired autophagosome-lysosome fusion or inefficient lysosome biogenesis and maturation. Autophagy is a very important biological phenomenon involved in regulating the metabolic balance between the synthesis, degradation and reuse of cellular substances [4]. Lysosomal protein degradation is the final step in the autophagic process, where *LAMP*2 located on chromosome Xq24 plays a key role in mediating the fusion of autophagosomes with lysosomes to form autolysosomes, and the causative defect of *LAMP*2 gene led to failure of cellular autophagy with accumulation of glycogen granules and intracytoplasmic vacuoles containing autophagic material [5, 6].

The typical clinical triad of the disease is HCM, skeletal myopathy, and mental retardation [7], but involvement of other organs is typical of the disease in males, and DD has been reported to cause retinal disease, liver disease and lung disease [8–10], suggesting that it is a multisystem disease. In contrast, female patients usually present with a milder phenotype, in which cardiac disease may act as an isolated clinical feature (73% of patients [11]), but it can be as severe as in males. The disease progresses rapidly to end-stage heart failure, and unless a heart transplant (HTx) is performed, males usually die by age 30, while female patients die at 40 or 50 years.

For cardiac involvement, HCM is a major determinant of clinical presentation and prognosis [12]. In males, cardiac symptoms usually begin in infancy/childhood or adolescence and death from heart failure occurs in the third decade. Female patients are less affected because of the later onset and slower progression of the disease [2, 13]. All patients with onset in infancy/childhood or adolescence have cardiac symptoms, with the first symptoms being chest tightness [14], heart murmur [15], palpitations, or fatigue [16]. All patients had an abnormal electrocardiogram (ECG) during the asymptomatic phase. The most common ECG abnormality was Wolff-Parkinson-White (WPW) syndrome (69% of patients) [17, 18], other ECG abnormalities included complete atrioventricular block (AVB), supraventricular tachycardia, delta waves, and high precordial voltage [13] also appear in some cases. Echocardiography always shows typical concentric left ventricular (LV) hypertrophy [19] with preserved ejection fraction (EF) [20] in the early stage of DD, but progressed toward dilated pattern later. Serum troponin-T (TnT) levels are elevated in the acute cardiac phase [21], especially those with rapidly fatal outcome [22]. Cardiac Magnetic resonance imaging (MRI) can be used to differentiate DD from non-ischemic cardiomyopathy [23], analyze HCM patterns [24], and monitor fibrosis progression, which is essential to identify implantable cardioverter defibrillator (ICD) implantation or HTx [25].

Regarding skeletal muscles invasion, for male patients, 80–100% suffer from skeletal myopathy [26], which is usually mild and rarely severe [27]. About 10% of male patients have fatigue, poor exercise tolerance, or myalgia [28]. A study showed that the proximal muscles of people with DD were 60% weaker than those of healthy males [29]. A small number of male patients present with motor delay [30], dysarthria [31], or dysphagia. For female patients, myopathy is rarely observed [32] and when is present, muscle weakness is usually mild (30% weaker in females with DD than in healthy females [29]). In DD patients with skeletal muscle involvement, serum creatine kinase (CK) levels are always elevated in males (3 to 35 times higher than normal [13]), and normal or mildly elevated in females (up to 2 times the normal level [33]).

Neurologically and cognitively, mild to moderate deficits are found in 70–100% of male patients [34], while significant mental retardation is rare [16], and these deficits include speech delay [14], attention deficits [10], and behavioral abnormalities. Other neurological symptoms, such as autism [30], psychosis and depression [35] are rarely reported. However, only 6% of female patients had mild mental retardation [36], but 50% had mild learning or cognitive impairment during childhood [37]. Unexplained neuropathy occurred 3 times more often in females than in males [38]. Positron emission tomography (PET) and MRI showed decreased cortical glucose metabolism [39] or central nervous system lesions.

Pathological findings include intracytoplasmic vacuoles containing autophagy and glycogen in skeletal and cardiac muscle cells [40]. The exact prevalence is unknown, and the incidence of DD is on the rise with the development of *LAMP*2 gene testing. We describe here a novel exonic mutation in the *LAMP*2 gene (c.1052delG) that results in coding shift and may lead to premature truncation of the mutant *LAMP*2 protein.

## Case presentation

**Fig. 1:**
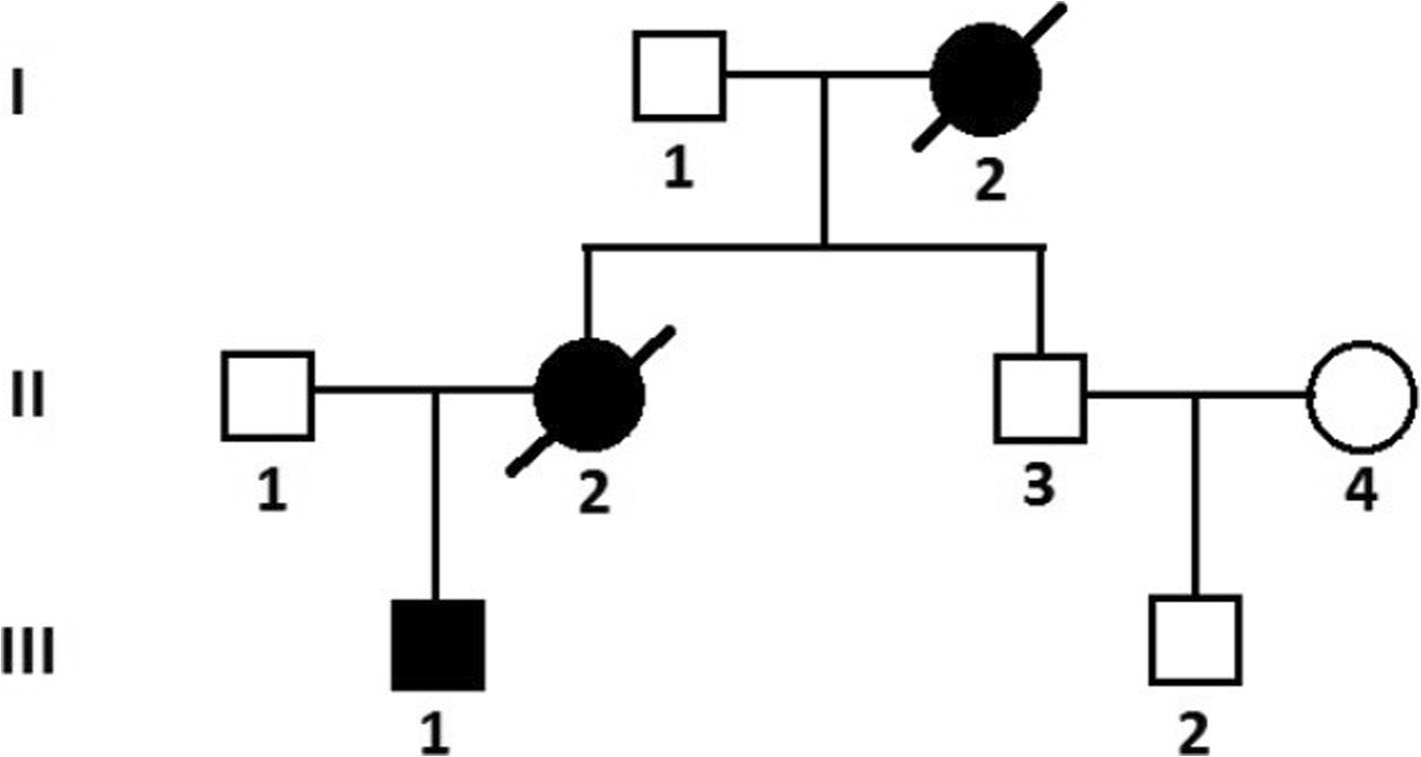
Pedigree of the family with lysosomal associated membrane protein-2 (*LAMP*2) mutation. III-1 is proband. Black symbols indicate affected subjects, white symbols indicate unaffected subjects. Slashed symbols indicate the deceased members

The proband (III-1), a male patient in early adolescence admitted to hospital on December 23, 2015 with heart failure (Fig. 1). He had a history of visual impairment and was diagnosed with highly myopic in elementary school, which was partially corrected with glasses. He also exhibited some mental retardation, learning and expressing difficulties, all of which were present in his kindergarten. At the time of his visit to the doctor, his intellectual level was only equivalent to the end of elementary school, even though he was 18 years old.

**Fig. 2:**
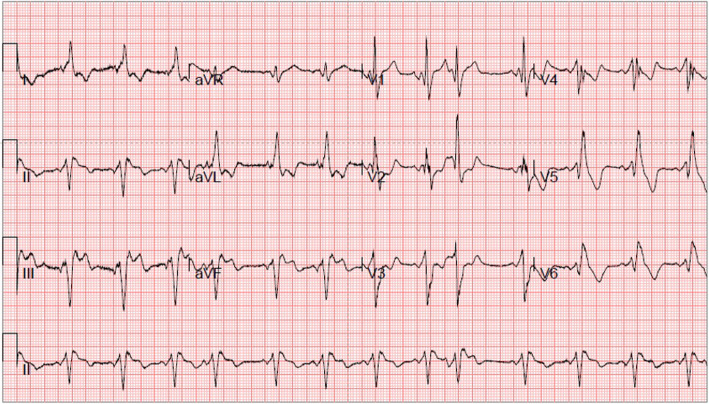
Electrocardiogram of the proband aged 18 years shows sinus rhythm, premature atrial contractions, Wolff-Parkinson-White (WPW) syndrome

**Fig. 3:**
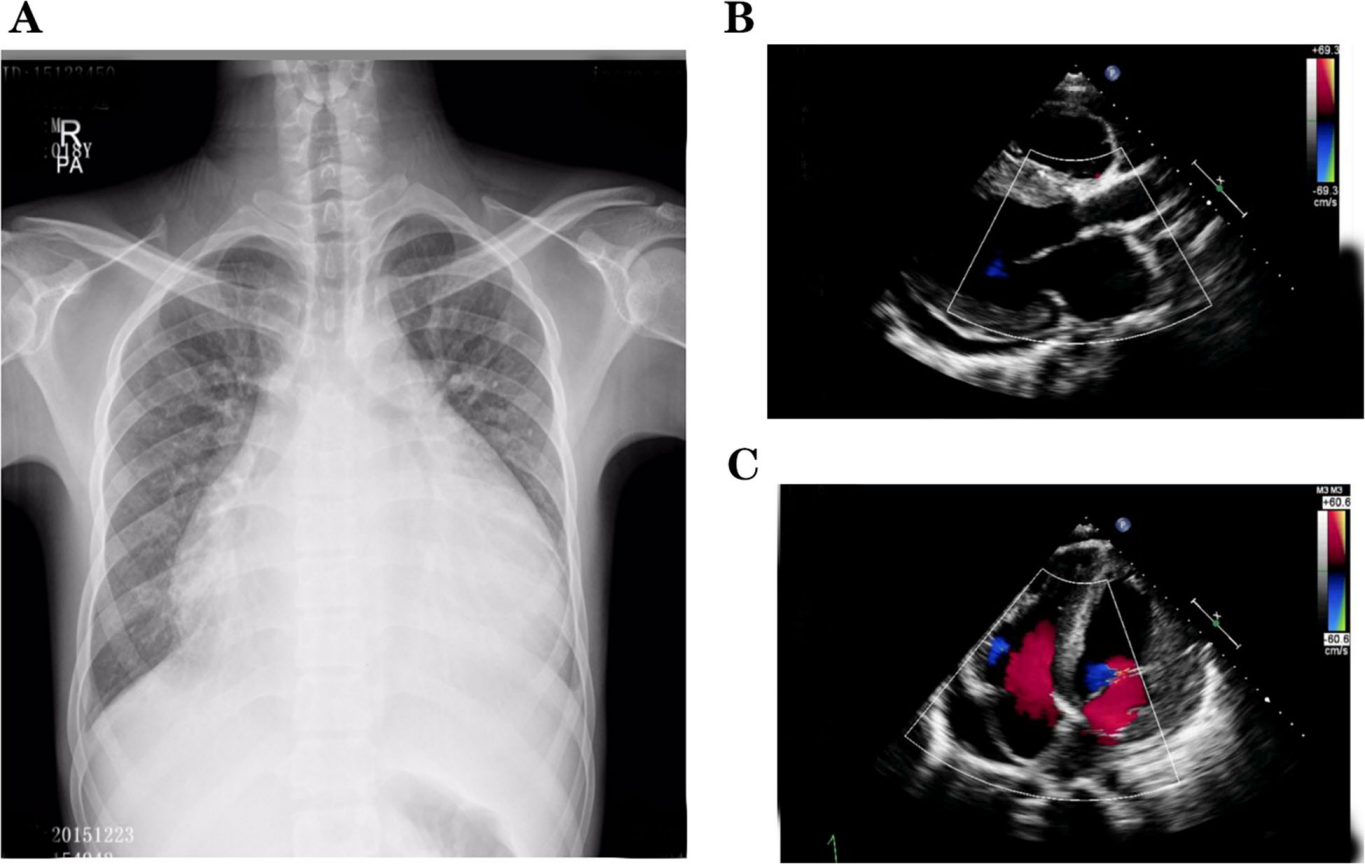
Chest radiographs and transthoracic echocardiography assessment of the proband. **a** Chest radiographs suggest bilateral enlargement of the heart. **b**, **c** Transthoracic echocardiography shows symmetrical hypertrophy, dilatation of all four cavities, and moderate amount of pericardial effusion

**Fig. 4:**
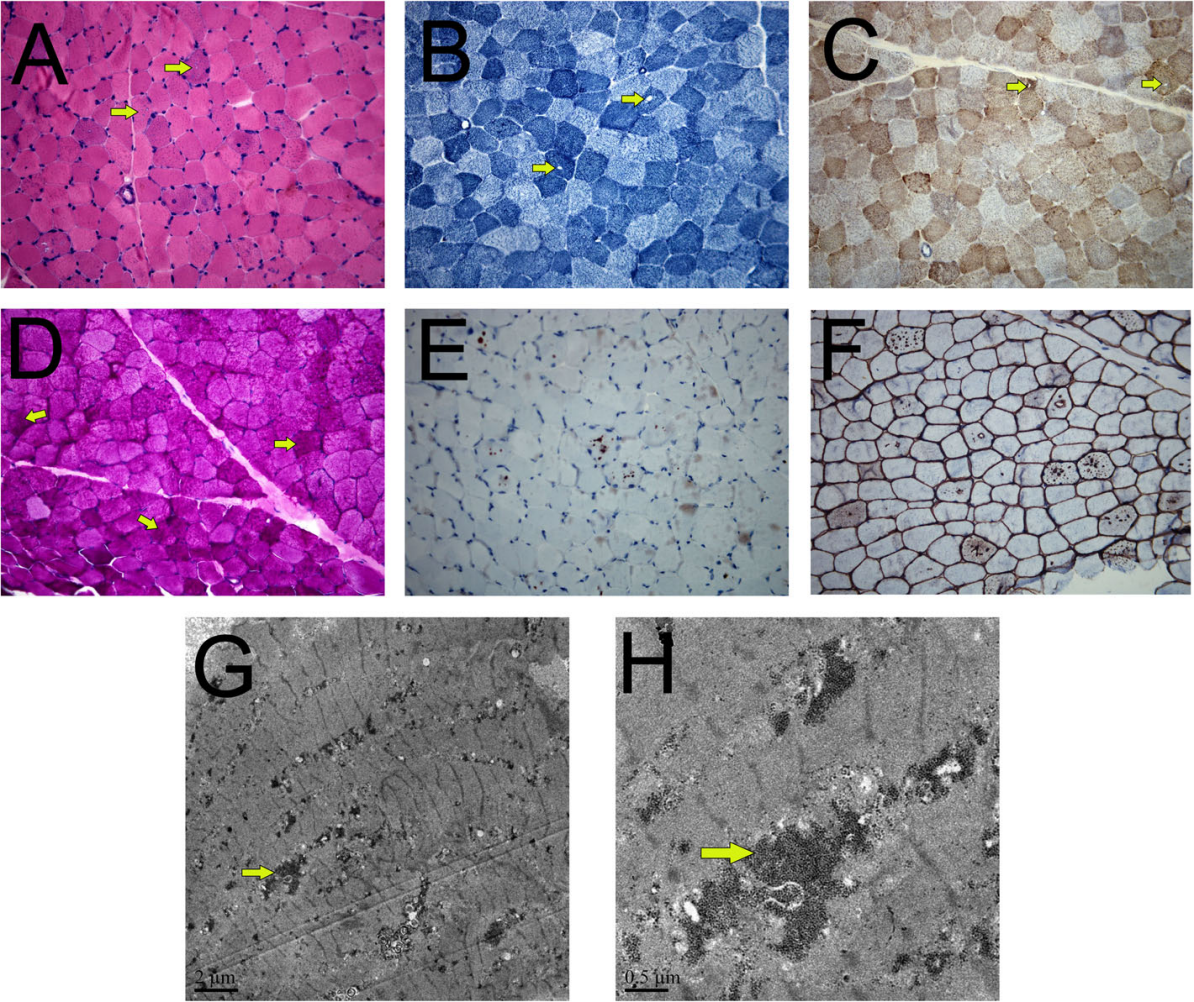
Histopathology analyses of the vastus lateralis muscle biopsy from the proband. HE staining showed small vacuoles within the muscle fibers containing basophilic particals (**a**, ×400); NADH-TR (**b**, ×400) and COX staining (**c**, ×400) demonstrated intracytoplasmic vacuoles in some myofibers; PAS staining (**d**,×400) showed abnormal glycogen granules deposition in muscle fiber vacuoles; Immunohistochemistry demonstrated only a few intracytoplasmic vacuoles displayed the expression of *LAMP*2 protein (**e**, ×400), and high expression of dystrophin both on myofiber membrane and in vacuoles (**f**, ×400); Electron microscopy revealed many glycogen particles deposited in the cytoplasm (**g**, **h**)

The main symptoms of the proband were recurrent chest tightness and asthma for 6 months after activity, and occasional palpitations and fatigue. One week after admission, the condition deteriorated to nocturnal paroxysmal dyspnea and anorexia. On the day of admission: physical examination revealed muscle atrophy of the proximal limbs and back. Biochemical examination on admission indicated: aspartate aminotransferase (AST), 289 U/L (normal range, 15–40 U/L); creatine kinase (CK), 732 U/L (38–174 U/L); CK-MB, 23 U/L (0–23 U/L). ECG showed sinus rhythm, atrial premature beat, WPW syndrome (Fig. 2). Chest X-ray suggested heart expands bilaterally (Fig. 3). Transthoracic echocardiography revealed left ventricular hypertrophy with dilatation of all four chambers, and diffuse decrease of wall movement: interventricular septum thickness diastolic (IVSTd), 15 mm; left atrial dimension (LAD), 46.5 mm; left ventricular end diastolic diameter (LVDd), 62 mm; pulmonary artery systolic pressure (PASP), 70 mmHg; left ventricular ejection fraction (LVEF), 24% (Fig. 3). In combination with the symptoms, physical examination, laboratory tests, and medical history of the proband, we suspected DD as the underlying cause. A biopsy of the lateral femoral muscle was performed to confirm this. Hematoxylin-eosin (HE) staining showed small vacuoles within the muscle fibers containing basophilic particles (Fig. 4a). NADH-TR staining (Fig. 4b) and COX staining (Fig. 4c) revealed the presence of intramural vacuoles in some muscle fibers. An unusual periodic acid-Schiff (PAS) staining was localized in the vacuoles, suggesting glycogen deposition (Fig. 4d). Immunohistochemical analyses using a commercial anti-human *LAMP*2 antibody demonstrated only a few intracytoplasmic vacuoles displaying the expression of *LAMP*2 protein (Fig. 4e). Furthermore, Anti-dystrophin staining showed expression of dystrophin protein in intracytoplasmic vacuoles within diseased muscle fibers (Fig. 4f). Electron microscopy revealed many glycogen particles deposited in the cytoplasm (Fig. 4g, h).

**Fig. 5:**
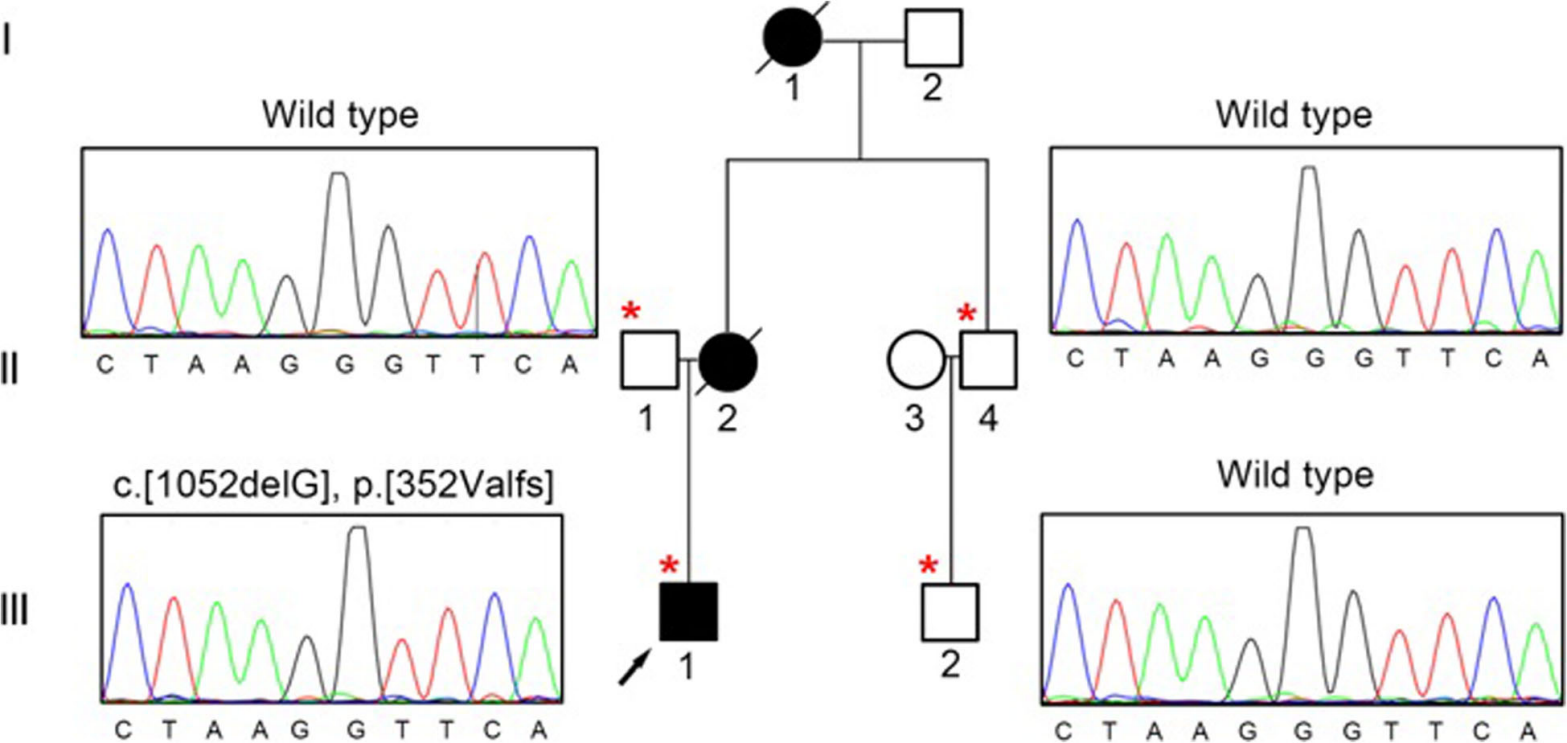
1052delG was detected by DNA sequence analysis of exon 8 of *LAMP*2. The DNA sequence of the proband (III-1) shows the hemizygous deletion

In terms of family genetic history, his mother (II-2) was diagnosed with ‘heart disease’ at the age of 24 and died suddenly 2 years later. His maternal grandmother (I-2) died of ‘heart disease’ at the age of 30. For genetic testing/mutations, Genomic DNA was extracted from the peripheral blood of the proband. A novel frameshift mutation referred to hemizygous mutation (c.1052delG) in exon 8 of *LAMP*2 (Fig. 5) was revealed which predicted to cause truncation of the protein. The same mutation was not found in his father, uncle (II-3) and cousin (III-2) whose electrocardiogram and echocardiography are normal.

Two months after discharge, the proband was readmitted with congestive heart failure and cardiogenic shock. Unfortunately, his family forewent invasive treatment and the proband died within 24 h of his second admission.

## Discussion

In recent years, the development of exome sequencing technology and bioinformatics has brought cost-effective and highly accurate results, which have been widely used in the diagnosis of genetic diseases [15]. Exome sequencing has been used to examine the genetic basis of familial HCM cases [41]. In this case, a novel code-shifting mutation, the hemizygous mutation (c.1052delG), was identified in exon 8 of *LAMP*2, which exhibits the HCM phenotype. In addition, among the family members suspected of carrying the *LAMP*2 mutation, only two female members were diagnosed with heart disease and one of them died suddenly. The proband had all of the above-mentioned clinical triad associated with DD, as well as cognitive and neurological deficits, but her father, uncle (II-3), and cousin (III-2) did not have the same mutation, and the electrocardiogram and echocardiogram were normal. These findings suggest that this genetic mutation is actually pathogenic and that the novel mutation of exon 8 of *LAMP*2 may be relevant in determining phenotype severity, earlier onset of heart failure, certain risk of sudden death, worse prognosis, and severe cognitive and neurological impairment.

Among the previously reported cases, we found that only one exception, the 928G > A mutation on exon 7, occurred in Africans [30], while all other cases were Asians and Caucasians. The reason could be the low prevalence in Africans or the infrequency of testing (e.g. genetic testing), leading to missed and misdiagnosis. Therefore, in this article, we only compared the mutant loci differences between Asians and Caucasians. By analyzing previous reports of DD mutations, we found that in all reported cases, the mutation sites in exon 2 were Caucasian, while most of the mutation sites in exon 6 were Asian (Table 1). However, we did not find a correlation between mutations and phenotypes. For example, the 928G > A mutation, which currently has the highest incidence, has been reported in many patients, but its phenotype varies: Bertini et al. [42] reported cases of learning and movement difficulties in infancy with a diagnosis of HCM at the age of 13. Arad et al. [43] reported a typical triad of DD; Burusnukul et al. [30] reported an unusual presentation of DD: autism, motor retardation, and normal cardiac evaluation. These different phenotypes of the same mutation illustrate the difficulty of determining the correlation between genotype and phenotype. However, Bottillo et al. [44] investigated the association between mutation types and clinical symptoms. They noted that missense mutations were associated with a lower incidence of cardiomyopathy; truncation mutations had the earliest onset, followed by splicing mutations, and missense mutations had the latest onset, which is consistent with the findings of D’souza et al. [17]. Similarly, Fu L et al. [15] suggested that truncating mutations may lead to earlier onset and more severe phenotypes. The truncating mutation in our study resulted in severe phenotypes, which corroborates the above view.

**Table 1:** Racial differences in the different exon mutations and the specific mutation sites for each exon mutation

	Asian (***n*** = 26)		Caucasian (***n*** = 26)		***P*** value
Exon 1	*n* = 2	29_35dupCGGGCTC	*n* = 1	52 T → C	1.00
		64G → T			
Exon 2	*n* = 0		*n* = 6	102_103delAG	**0.023**
				121delT	
				135dupA	
				137G → A	
				138G → A	
				179delC	
Exon 3	*n* = 6	189_190delTG	*n* = 2	294G → A	0.248
		241delG		294G → A	
		257_258delCC			
		317_320dupCATA			
		320_321insCATC			
		369_370delTG			
Exon 4	*n* = 1	467 T → G	*n* = 3	453delT	0.610
				467 T → G	
				605C → G	
Exon 5	*n* = 4	573delA	*n* = 2	680_701del	0.668
		718C → T		716delT	
		718C → T			
		741 + 1G → T			
Exon 6	*n* = 7	749C → G	*n* = 1	796_797insC	**0.049**
		749C → A			
		808dupG			
		808dupG			
		808_809insG			
		821_822delTT			
		839delA			
Exon 7	*n* = 1	877Cys → Thr	*n* = 5	928G → A	0.191
				928G → A	
				928G → A	
				928G → A	
				928G → A	
Exon 8	*n* = 2	973dupC	*n* = 6	940delG	0.248
		1009_1010delGT		961 T → C	
				962G → A	
				973insC	
				1075C → T	
				Single nucleotide delete	
Exon 9	*n* = 3	1204A → T	*n* = 0		0.235
		1205delC			
		1205delC			

The chi-square test was used for statistical analysis

Bold values indicate significance at *p*-value < 0.05

HCM is a major determinant of clinical presentation and prognosis [12]. DD is often misdiagnosed as HCM in the early symptomatic stage, leading to inappropriate treatment and further deterioration of the disease [45]. By analyzing the clinical manifestations of DD in previous literature, we found that serum creatine kinase (CK) was elevated in more than 90% of DD patients [17]. Based on this finding, we recommend screening for *LAMP*2 mutations in patients with a diagnosis with HCM with elevated serum CK. The ocular symptoms of DD are now receiving increasing attention [46]. By reviewing all case reports to date, we found that 60% of patients with DD had ocular involvement, including strabismus, myopia, and retinopathy [47]. Cardiomyopathy has been reported as the only clinical manifestation in women [36, 48]. Therefore, although clinical assessment is helpful in speculating about DD, genetic analysis is the gold standard for determining the etiology of HCM [24]. This information is essential to determine appropriate therapeutic strategies and to determine the genetic risk of family members. Therefore, sequence analysis is of great importance in the clinical differential diagnosis of DD [15].

The current therapeutic interventions for DD are mainly through ICD implantation and HTx to prevent and counter sudden death and heart failure. Because recent studies suggest that the underlying mechanism of DD is the blockade of autophagy, new drugs associated with autophagy, including autophagy activators and inhibitors, may be used in the near future to design new intervention strategies.

## Data Availability

Data archiving is not mandated but data will be made available on reasonable request.

## Abbreviations

LAMP2: Lysosomal-associated protein-2
NGS: Next-generation sequencing
DD: Danon disease
HTx: Heart transplant
WPW: Wolff-parkinson-White
HCM: Hypertrophic cardiomyopathy
ECG: Electrocardiogram
MRI: Magnetic resonance imaging
PET: Positron emission tomography
ICD: Implantable cardioverter defibrillator
LVEF: Left ventricular ejection fraction
